# China's country image in the eyes of international students from central Asian countries

**DOI:** 10.3389/fpsyg.2022.569789

**Published:** 2022-10-05

**Authors:** Xueyan Zhao, Xuqun You, Shanyan Lin

**Affiliations:** ^1^School of Educational Science, Yili Normal University, Yining, China; ^2^School of Psychology, Shaanxi Normal University and Shaanxi Provincial Key Laboratory of Behavior and Cognitive Neuroscience, Xi'an, China; ^3^Shaanxi Normal University, Xi'an, China

**Keywords:** China's country image, conceptualization, measurement, central Asian countries, effects

## Abstract

This study aimed to investigate the conceptualization and measurement of China's country image (CI) as well as its effects on the economic image, product image, national image and residence intention of individuals. A total of 297 international students from central Asia were recruited to complete an online survey. The confirmatory factor analysis of CI scale showed that CI is a multidimensional construct consisting of a cognitive component (further be divided into government image, international image, and social image) and an affective component. Participants' ratings on China's CIs before and after coming to China were compared by using a repeated-measures ANOVA and paired sample *t*-tests, and the results showed a significant difference in government image and international image but no significant difference in social image. The regression analysis showed that CI significantly predicted the economic image, product image, national image and individual's residence intention. This study demonstrated a theoretical insight of CI research and could potentially contribute to optimizing strategies to improve the country reputation.

## Introduction

Nowadays, the continuing globalization and productivity are accelerating communication and collaboration among countries worldwide. Country image (CI) has been regarded as a critical form of soft power in this situation. CI is also considered as one of the political and economic capitals in a nation, which reflects a country's standing in the international arena (Wang, 2006). Therefore, every country has, more than ever before, been concerned with its own international image to promote global reputation and to facilitate active cross-cultural communications. Moreover, CI could also contribute to a multitude of potential economic, cultural and political benefits (Chen et al., 2020). Taking these advantages into account, lots of scholars and practitioners have emphasized the significance of CI in understanding and appraising a given country from different perspectives of disciplines. Therefore, the increased importance of CI has stimulated large number of studies in a wide range of scientific fields (Ingenhoff et al., 2019).

Since 1960s, much effort in academia has been devoted to better understanding the construct of CI, as well as its effects from a multidisciplinary perspective (Nagashima, 1970; Min Han, 1989; Roth and Romeo, 1992; Martin and Eroglu, 1993; Li et al., 2014; Carneiro and Faria, 2016; Lee et al., 2016; Lu et al., 2019; Mikhnevych et al., 2020; Dragoi, 2021; Gohary et al., 2022). However, most of the previous studies were just descriptive/qualitative studies, especially in China (Sun, 2002; Shao, 2014; Liu, 2016; Li et al., 2021; Gohary et al., 2022). For example, many scholars have conducted an in-depth analysis of the importance and significance of China's CI with event report to formulate countermeasures (Feng, 2008; Xue et al., 2015; Chen et al., 2020), but little attention has been paid to the integrated and systematic analysis of conceptualization, components, and impacts of CI from interdisciplinary perspectives, such as the research on the relationship between country image and international education. Although some studies have attempted to determine the structural and psychometric properties of China's CI in a quantitative way, no consistency has been reached so far on the components and dimensions of CI. Furthermore, the findings regarding the impacts of CI and its robustness appeared inconsistent (Wang et al., 2012; Lei et al., 2015). Accordingly, there is an urgent and challenging task to describe and examine the structural and psychometric model of China's CI, especially adopting sound statistical approach to provide new and valuable insights. Thus, this study aims to develop a promising conceptualization and measurement model of China's CI and examine the effects of CI on the economic image, product image, national image, and residence intention of international students from central Asian countries.

The central Asian countries along the Silk Road (mainly including Kazakhstan, Kyrgyzstan, Tajikistan, Uzbekistan, and Turkmenistan) have a long history of political, economic, and cultural exchange with China. Therefore, it is important for China to maintain good CI as it contributes greatly to the development of mutual friendships and bilateral/multilateral cooperation between countries. Clearly, there is a need to gain more knowledge about China's CI for individuals from central Asian countries. For this purpose, a questionnaire survey is conducted in this study with international students from central Asian countries who are currently studying or have previously studied in China for some time, as international students are, by definition, the population group with the highest mobile and they are the main audience to communicate the images of their host countries.

This study aims to (1) provide an integrated conceptual framework of CI based on previous studies; (2) quantitatively determine the construct of CI toward China; (3) compare China's CI in the eyes of international students from the central Asian countries before and after their coming to China; and (4) examine the effects of CI on product image, economic image, national image, and residence intention. The study not only put forward a theoretical insight of CI's conceptualization and construction, but also provides practical reference in optimizing the image of China to enhance worldwide understanding.

## Literature review and research hypotheses

### Conceptualization and measurement of CI

Despite the acknowledged importance of CI, there is no generally accepted conceptualization of CI (Lu et al., 2019). Bannister and Saunders (1978, p. 562) described CI as “generalized image, created by variables such as representative products, economic and political maturity, historical events and relationships, traditions, industrialization, and the degree of technological virtuosity.” In a similar vein, Martin and Eroglu (1993, p. 193) defined CI as “the total of all descriptive, inferential, and informational beliefs one has about particular country.” Apparently, both of them highlighted the cognitive characteristics of CI from a static perspective. However, Verlegh and Steenkamp (1999, p. 525) defined CI as “mental representations of a country's people, products, culture, and national symbols of a country.” Meanwhile, Verlegh (2001, p. 25) stated that “a mental network of affective and cognitive associations connected to the country.” In China, Sun (2002, p. 16) argued that CI was an evaluative impression individuals held inside and outside of the country, which was concerned with politics (e.g., government, diplomacy, and military), economics (e.g., financial strength, fiscal solvency, product quality, and citizen income), society (e.g., social cohesion, security and stability, and national morale), culture (e.g., scientific and technological strength, education level, cultural heritage, customs, and values), and geography (e.g., geographical environment, natural resources, and population size).

Taken together, CI in this study is considered as an overall perception and evaluation an individual holds about a country, and it is an integrated mental representation of various aspects of a country such as political maturity, economic prospects, industrialization and degree of technical virtuosity, products, historical events, diplomatic relationship, culture, traditions, and social life.

According to the conceptualization of CI, some scholars have underscored the cognitive representation of CI (Gertner and Kotler, 2004). Guo (2017) measured China's cognitive CI including political, economic, cultural, social, and ecological image according to Sun's (2002, p. 16) conceptualization, but many scholars argued that the construct of CI is composed of at least a cognitive component and an affective component (Li et al., 2014; De Nisco et al., 2015; Hwang et al., 2020). Some scholars have found that emotion can lead to much stronger psychological reactions than cognition, and thus, it is necessary to particularly consider the affective component of CI (De Nisco et al., 2015). In China, Ma's (2014) contribution was concerned about both of them.

Based on the conceptual underpinnings of the attitude theory (Fishbein and Ajzen, 1977), CI is composed of a cognitive component, an affective component, and a conative component (Parameswaran and Pisharodi, 1994; Laroche et al., 2005). The cognitive component consists of multiple beliefs and evaluations of all aspects of a country such as the government, military, environment, culture, education, media, and the living standard (Heslop and Papadopoulos, 1993; Li et al., 2014) the affective component comprises of affective feelings and responses regarding the country, while the conative component refers to the intended behavior (Roth and Diamantopoulos, 2009), such as the intention or willingness to visit or live in the country and socialize with locals. However, some scholars have argued that the intended behavior should be viewed as an outcome of cognitive and affective CI rather than the component of CI (Roth and Diamantopoulos, 2009; Li et al., 2014). In addition, it is also important to distinguish the effects of cognitive and affective components, as the affective component may lead to much stronger effects than the cognitive component (Nebenzahl et al., 2003; Li et al., 2014). Thus, CI has been shown to be a multidimensional construct (Lala et al., 2008; Hakala et al., 2013).

Notwithstanding, Kleppe et al. (2002) pointed out that individuals with limited knowledge of a country could also develop positive CI toward this country. Image measurement is, therefore, a useful strategic tool for branding a nation (Echtner and Ritchie, 1993; Carneiro and Faria, 2016). Accordingly, it is necessary to develop a valid and reliable measurement instrument of CI to obtain better understanding of the construct and its effects (Papadopoulos, 2004; Magnusson and Westjohn, 2011; Buhmann and Ingenhoff, 2015a). However, controversy remains about the definition and conceptualization of CI (Roth and Diamantopoulos, 2009; Newburry, 2012). Meanwhile, the measurement instrument of CI especially for China's country image is lacking, and it is necessary to develop an effective tool to clarify the presentation and elements of CI according to the previous studies (Ma, 2014; Guo, 2017).

Thus, based on the literature about conceptualization and measurement of CI, this study proposed the first hypothesis:

H1: All these components of CI can be divided into four core components: an affective component and three cognitive components (including government image, social image, and international image).

In addition, the CI may change with the development of the country, and it also shows considerable intraindividual variability with one's direct or indirect experience toward the country. However, it is also noted that CI is a mental representation which is a relatively stable result of long-term communication. Thus, it is a challenging but valuable issue to determine the characteristics of country image. In this study, we examine CI's nature of dynamism and relative stability through pairwise comparisons of international students' beliefs and evaluations of China's CI before and after coming to China. Thus, this study would test the following hypothesis:

H2: There is a difference of China's CIs in the eyes of the international students from central Asian countries before and after their coming to China.

### Effects of CI and related variables

Previous studies indicated that the CI, as the mental representations of the people, products, culture, and national symbols of a country, has significant effects on consumers' evaluation of products and purchase intention (Askegaard and Ger, 1997; Roth and Diamantopoulos, 2009; Li et al., 2014). Verlegh and Steenkamp (1999) found that CI acted not only as a cognitive cue but also as symbolic and emotional meaning to consumers, and thus, it had more significant effects on perceived quality than on attitude toward the products or purchase intention. Thus, CI can serve as an important antecedent of behaviors. However, the research on the effects of CI is rather limited and mainly highlighted the effects of CI from theoretical analysis in value orientation, so there is a need to accurately determine the effects of CI on other variables with quantitative approach.

CI can also have significant effects on investment, visiting, and residence intention (Verlegh and Steenkamp, 1999; Yu et al., 2015). A recent review shows that CI has important economic, cultural, and political consequences (Buhmann and Ingenhoff, 2015b). In addition to the role as the informational cue, CI also has strong emotional and affective connotations formed *via* direct or indirect experiences, which in turn can affect individuals' behavioral intention (Verlegh and Steenkamp, 1999).

It is also necessary to clarify the distinction between CI and product image (PI), economic image (EI), and national image (NI) (Li et al., 2014; Carneiro and Faria, 2016). CI is the integrated impression toward a country, while the other three concepts focus mainly on a specific aspect of the country (Erickson et al., 1984; Han et al., 1994). For example, PI refers to consumers' general perceptions or beliefs of a country's products, such as technology advancement, workmanship, and serviceability (Roth and Romeo, 1992; Li et al., 2014); EI captures the development scale, potential, and prospect of a country (Buhmann, 2016; Jia-xun et al., 2017), while NI relates to the people of a country with positive or negative characteristics such as honesty, friendliness, or selfishness (Ma, 2014; Guo, 2017). Therefore, CI is probably an independent construct with some associations with PI, EI, and NI (e.g., Parameswaran and Pisharodi, 1994; Castano et al., 2016). In sum, CI is an umbrella construct (Roth and Diamantopoulos, 2009; Elliot and Papadopoulos, 2016), which also plays an important role in individual's evaluations and behaviors (Li et al., 2014). Wang et al. (2012) discussed that CI and PI were distinct but associated with each other in addition to having different influences on individual's evaluations and their purchase intentions.

In addition, the beliefs and attitudes toward a country do not stay unchanged overtime. Instead, they vary with one's direct or indirect experience of the country's politics, economy, and culture. Given the acknowledgment of the halo effects of CI (Kotler and Gertner, 2002; Josiassen et al., 2013), this study also aims to extend our current understanding of the conceptualization of CI and its potential effects on PI, EI, NI, and individual's residence intention.

Thus, this study would test the following hypothesis:

H3: The overall CI can predict national image, product image, economic image, and residence intention.

## Methods

### Participants and procedures

In this study, a total of 297 international students from central Asian countries were recruited to complete a self-administered online survey through a network platform *wenjuanwang*. Of these 297 participants, 136 were from Kazakhstan, 85 were from Kyrgyzstan, 41 were from Tajikistan, and 35 were from Uzbekistan. All participants were studying or had studied in China for more than half a year. There was a relatively even distribution of gender (51.2% female and 48.8% male), and the most majority of participants (79.5%) were aged 18–35 years.

### Measures and instruments

*Overall Country Image Scale* consisted of three cognitive components (17 items) and one affective component (three items). The cognitive component included government image (five items), international image (five items), and social image (seven items). All items were adapted from previous literature (Ma, 2014; Guo, 2017). All participants were instructed to evaluate their impressions to each item before and after coming to China on a five-point scale ranging from 1 (*very poor*) to 5 (*very good*). In this study, Cronbach's alpha coefficients ranged from 0.72 to 0.83 for all subdimensions, and the overall Cronbach's alpha coefficient for the whole scale was 0.91 and 0.92 for before and after coming to China, respectively, indicating that the scale had acceptable reliability. All the loadings of items to factors were above 0.5 (range: 0.51–0.82), indicating strong association between the factors and their respective items.

*Product Image Scale* was a six-item scale for measuring individuals' perception of product quality, technology, price, service, and function (Li et al., 2014). All items were rated on a five-point scale ranging from 1 (*very poor*) to 5 (*very good*). In this study, Cronbach's alpha coefficient was 0.71, indicating acceptable reliability. The correlations of items to factors ranged from 0.46 to 0.65.

*Economic Image Scale* was used to measure the scale, speed, and potential of China's economy development (three items). The items were rated on a five-point semantic differential scale ranging from 1 (*very inaccurate*) to 5 (*very accurate*). In this study, Cronbach's coefficient was 0.79, indicating satisfactory reliability. The correlations of items to factors were above 0.5 (range: 0.62–0.87).

*National Image Scale* was comprised of 12 items adapted from previous literature (Ma, 2014; Guo, 2017) and interviews of five overseas students, which was used to measure positive national image (seven items) and negative national image (five items). Items were rated based on a bipolar, entirely verbalized five-point Likert scale ranging from 1 (*very inaccurate*) to 5 (*very accurate*). Cronbach's alpha was 0.82 for the positive national image subscale and 0.78 for the negative national image subscale, suggesting satisfactory reliability. The correlations of items to factors were above 0.5 (range: 0.51–0.80).

*Residence intention* was measured using a single item.

Some demographic variables were considered, including citizenship, gender, age, educational degree, duration in China, and Chinese proficiency. All the items were presented in Russian because all participants were proficient in Russian, which were checked by four experts from four different central Asian countries. All items and construct reliabilities are shown in **Table 3**. For the requirements of the comparative analysis of China's CI, all items were scored twice in this cross-section study according to the experiences of the international students from central Asian countries before and after their coming to China.

## Results

### Evaluation of the measurement model

Before further analysis of interrelationships of these subscales, confirmatory factor analysis (CFA) was conducted using Mplus 7.0. The appropriateness of the measurement model was examined using a set of absolute and relative fit indexes (Hu and Bentler, 1999) such as comparative fit index (CFI), Tucker–Lewis index (TLI), the ratio of chi-square to degrees of freedom (χ^2^/df), root mean square residual (RMSR), and root mean square error of approximation (RMSEA). As shown in Table 1, the measurement models of the four subscales had adequate reliability and convergent validity, and all items showed significant loadings on their respective factors (*p* < 0.001) with values higher than 0.50. Table 2 shows that all indexes were within the recommended range, indicating a good fit (Marsh et al., 2004). The composite reliability and homogeneity coefficient (Bentler, 2009; Rios and Wells, 2014) of the overall country image scale were 0.94 and 0.84, respectively.

**Table 1:** The items loadings (λ) and coefficients (α) of measurement models.

**Scales**			**Before coming to China**		**After coming to China**	
	**Factors**	**Items**	**λ**	**α**	**λ**	**α**
Cognitive component	Government image	Integrity	0.52	0.83	0.57	0.83
		Efficiency	0.81		0.75	
		Humanism	0.82		0.69	
		Leadership	0.73		0.78	
		Trustworthiness	0.66		0.74	
	International image	humanistic quality	0.61	0.72	0.63	0.75
		Technology	0.52		0.59	
		Education	0.63		0.63	
		Military	0.62		0.52	
		Diplomatic	0.79		0.64	
	Social image	Food safety	0.62	0.82	0.59	0.82
		Urban hygiene	0.67		0.63	
		Natural environment	0.59		0.58	
		Social security	0.66		0.68	
		Air quality	0.51		0.55	
		Transportation	0.73		0.61	
		Films and TV	0.53		0.69	
Affective component		Favorability	0.56	0.75	0.74	0.81
		Reliability	0.68		0.79	
		World influence	0.64		0.69	
Reliability			0.91		0.92	
Economic image		Great scale	0.62	0.79		
		Grow up quickly	0.87			
		Great potential	0.75			
Product image		Quality	0.46	0.71		
		Technology	0.61			
		Price	0.56			
		Service	0.65			
		Function	0.47			
National image	Positive National image	Trustworthy	0.62	0.82		
		Warm and friendly	0.74			
		Politeness	0.64			
		Neat	0.51			
		Thoughtfulness	0.65			
		Helpful	0.62			
		Intelligent	0.60			
	Negative National image	Selfish	0.80	0.78		
		Intelligent	0.61			
		Conservatism	0.51			
		Arrogant	0.64			
		Rude	0.58			

**Table 2:** The results of coefficient α and indicators of CFA in subscales.

	**coefficient α**	**χ^2^**	**Df**	**χ^2^/df**	**CFI**	**TLI**	**RMSEA**	**RMSR**
BCI	0.91	373.31	161	2.32	0.92	0.90	0.07	0.05
ACI	0.92	347.07	158	2.20	0.93	0.91	0.06	0.05
EI	0.79	0.00	0	0.00	1.00	1.00	0.00	0.00
PI	0.71	2.88	2	1.44	1.00	0.99	0.04	0.01
NI	0.52	97.69	50	1.95	0.96	0.94	0.06	0.04
PNI	0.82	29.03	12	2.42	0.97	0.95	0.07	0.03
NNI	0.78	4.79	4	1.20	1.00	1.00	0.03	0.02

BCI, the overall country image of before coming to China; ACI, the overall country image of before coming to China; EI, economic image; PI, product image; NI, national image; PNI, positive national image; NNI, negative national image.

In addition, we tested the common method bias through Harman' single-factor test and controlling for the effects of an unmeasured latent methods factor with bi-factor model (Rodriguez et al., 2016). The result of the principle analysis indicated that loading all items on one factor explained 38.7% of the variance less than the cutoff of half (50%). The model fit indices of bi-factor model showed non-significant difference between five-factor model and five-factor model plus method (see Table 3). Therefore, these results demonstrated that the common method bias is unlikely to be a serious issue.

**Table 3:** The tests of divergent validity and common method bias to variables.

**Model**	**χ^2^**	**df**	**χ2/df**	**RMSEA**	**CFI**	**TLI**	**SRMR**
Single-factor model	2,796.83	740.00	3.78	0.10	0.57	0.55	0.10
Two-factor model	2,325.73	739.00	3.15	0.09	0.67	0.65	0.08
Three-factor model	2,120.00	737.00	2.88	0.08	0.71	0.70	0.08
Four-factor model	1,911.51	734.00	2.60	0.07	0.76	0.74	0.07
Five-factor model	1,679.50	730.00	2.30	0.07	0.80	0.79	0.07
Five-factor + Method model	1,468.58	691.00	2.13	0.06	0.84	0.82	0.12

Single-factor model = CI2 + EI + PI + PNI + NNI; Two-factor model = CI2, EI + PI + PNI + NNI; Three-factor model = CI2, EI, PI + PNI + NNI; Four-factor model = CI2, EI, PI, PNI + NNI; Five-factor model = CI2, EI, PI, PNI, NNI; CI2, the overall country image of after coming to China; EI, economic image; PI, product image; PNI, positive national image; NNI, negative national image.

### Preliminary analyses and descriptive statistics

The descriptive statistics of all subscale's items are shown in Tables 4, 5. Notably, the averages of most items exceeded three, the middle point of the scale.

**Table 4:** Descriptive statistics.

**Scale**	**Items**	***M***	***SD***
Economic image	Great scale	3.85	0.91
	Grow up quickly	4.12	0.95
	Great potential	4.08	0.91
Product image	Quality	3.47	1.15
	Technology	3.84	1.23
	Price	3.60	1.18
	Service	3.40	1.20
	Function	3.82	1.74
National image	Trustworthy	3.09	0.96
	Warm and friendly	3.64	0.97
	Politeness	2.98	0.95
	Neat	2.38	0.94
	Thoughtfulness	3.20	0.92
	Helpful	3.09	1.06
	Intelligent	3.55	0.95
	Selfish	2.92	1.00
	Indifference	3.01	1.01
	Conservatism	3.14	0.82
	Arrogant	2.71	0.96
	Rude	2.69	0.94

**Table 5:** Paired sample *T*-test of overall country image scale.

	**Before coming to China**		**After coming to China**			**95% Confidence interval of the difference**		***T***	***p***
**Items**	***M_1_***	***SD_1_***	***M_2_***	***SD_2_***	***D***	**Lower**	**Upper**		
Integrity	4.12	1.16	4.23	0.99	0.11	0.25	−0.03	1.49	0.14
Efficiency	4.15	1.07	4.36	0.89	0.21	0.32	0.10	3.71	0.00
Humanism	3.79	1.22	3.88	1.15	0.08	0.21	−0.04	1.29	0.20
Leadership	4.14	1.04	4.36	0.95	0.22	0.32	0.12	4.25	0.00
Trustworthiness	4.02	1.15	4.16	1.14	0.14	0.26	0.02	2.28	0.02
National quality	3.63	1.18	3.51	1.24	−0.12	0.04	−0.28	−1.51	0.13
Technology	4.37	0.86	4.64	0.70	0.26	0.36	0.16	5.07	0.00
Education	4.08	1.00	4.37	0.87	0.28	0.41	0.16	4.53	0.00
Military	4.01	1.18	4.29	1.01	0.28	0.38	0.18	5.46	0.00
Diplomatic	4.11	1.07	4.30	0.92	0.19	0.29	0.09	3.79	0.00
Films and TV	3.55	1.26	3.84	1.11	0.29	0.43	0.16	4.26	0.00
Food safety	3.35	1.26	3.20	1.20	−0.15	−0.01	−0.30	−2.14	0.03
Urban hygiene	3.48	1.30	3.37	1.27	−0.11	0.06	−0.28	−1.30	0.20
Natural environment	3.78	1.21	3.97	1.11	0.19	0.34	0.05	2.58	0.01
Social security	3.92	1.12	3.95	1.15	0.03	0.17	0.11	0.42	0.67
Air quality	3.10	1.30	2.61	1.24	−0.49	−0.33	−0.65	−6.00	0.00
Transportation	3.79	1.17	4.10	1.07	0.31	0.46	0.17	4.28	0.00
Favorability	4.00	1.11	4.40	0.88	0.40	0.53	0.28	6.34	0.00
Reliability	3.70	1.21	3.98	1.13	0.28	0.40	0.16	4.59	0.00
World influence	4.27	0.92	4.37	0.86	0.10	0.19	0.02	2.38	0.02
Overall impression	3.97	1.07	4.35	0.87	0.38	0.50	0.26	6.25	0.00
**Factor**									
Government image	4.04	0.87	4.20	0.79	0.15	0.24	0.07	3.51	0.00
Social image	4.03	0.74	4.24	0.68	0.01	0.09	−0.08	0.14	0.89
International image	3.65	0.81	3.63	0.79	0.18	0.26	0.10	4.34	0.00
Affective image	3.99	0.89	4.25	0.82	0.26	0.35	0.18	5.94	0.00
**Overall CI**	3.87	0.7	3.99	0.67	0.13	0.20	0.05	3.46	0.00

D = M_1_-M_2_.

### Comparison of CI before and after coming to China

Paired sample *t*-test was used to compare CI before and after coming to China. Table 5 shows that there were significant differences in most items (*p* ≤ 0.03). There were also significant differences in government image (*t* = −3.51, *p* < 0.01), international image (*t* = −4.34, *p* < 0.01), and affective component (*t* = −5.94, *p* < 0.01), but no significant difference in social image (*t* = −0.14, *p* > 0.05). It was also found that participants' CI before coming to China was significantly lower than that after coming to China (*t* = −3.47, *p* < 0.01). No significant changes were found for two items of government image (integrity and humanism), one item of international image (national quality), and two items of social image (urban hygiene and social security) (*p* > 0.05). However, participants' perceptions of food safety (*t* = 2.14, *p* < 0.05) and air quality (*t* = 6.00, *p* < 0.01) were significantly decreased after coming to China.

### The halo effects of overall CI

The correlation analysis suggested that each dimension was significantly related to the mean score of CI, as shown in Table 6. The overall CI before coming to China was only significantly related to positive national image (*r* = 0.20, *p* < 0.01), and the overall CI after coming to China was significantly related to positive national image (*r* = 0.50, *p* < 0.01), negative national image (*r* = −0.31, *p* < 0.01), economic image (*r* = 0.24, *p* < 0.01), and product image (*r* = 0.19, *p* < 0.01). The affective component of CI after coming to China was significantly related to positive national image (*r* = 0.51, *p* < 0.01), negative national image (*r* = −0.29, *p* < 0.01), economic image (*r* = 0.34, *p* < 0.01), and product image (*r* = 0.16, *p* < 0.01), while the affective component of CI before coming to China was significantly related to positive national image (*r* = 0.24, *p* < 0.01). Positive national image was significantly related to economic image (*r* = 0.37, *p* < 0.01) and product image (*r* = 0.26, *p* < 0.01). Economic image was significantly associated with product image (*r* = 0.21, *p* < 0.01).

**Table 6:** The means, standard deviations, and correlations.

	**M**	**SD**	**BGI**	**BSI**	**BII**	**BAC**	**AGI**	**ASI**	**AII**	**AAC**	**BCI**	**ACI**	**PNI**	**NNI**	**EI**
BGI	4.04	0.87	1												
BSI	3.57	0.85	0.480**	1											
BII	4.04	0.78	0.663**	0.625**	1										
BAC	3.99	0.89	0.583**	0.584**	0.609**	1									
AGI	4.20	0.79	0.596**	0.247**	0.348**	0.431**	1								
ASI	3.57	0.77	0.429**	0.594**	0.488**	0.511**	0.551**	1							
AII	4.22	0.68	0.436**	0.333**	0.532**	0.438**	0.685**	0.649**	1						
AAC	4.25	0.82	0.478**	0.323**	0.389**	0.601**	0.714**	0.629**	0.692**	1					
BCI	3.87	0.70	0.806**	0.856**	0.862**	0.786**	0.467**	0.617**	0.506**	0.507**	1				
ACI	3.99	0.67	0.541**	0.422**	0.487**	0.544**	0.847**	0.854**	0.868**	0.849**	0.584**	1			
PNI	3.13	0.67	0.203**	0.094	0.194**	0.244**	0.400**	0.394**	0.406**	0.508**	0.203**	0.499**	1		
NNI	2.89	0.69	−0.128*	0.041	−0.058	−0.067	−0.304**	−0.175**	−0.275**	−0.293**	−0.050	−0.307**	−0.395**	1	
EI	4.02	0.77	0.117*	−0.064	0.087	0.101	0.203**	0.121*	0.219**	0.342**	0.052	0.241**	0.373**	−0.108	1
PI	3.62	0.89	0.017	0.043	0.071	0.029	0.112	0.160**	0.225**	0.155**	0.048	0.186**	0.257**	−0.073	0.207**

*p < 0.05.

**p < 0.01.

BGI, government image of before coming to China; BSI, social image of before coming to China; BII–international image of before coming to China; BAC, affective component of country image before coming to China; AGI, government image of after coming to China; ASI, social image of after coming to China; AII, international image of after coming to China; AAC, affective component of country image before after coming to China; BCI, overall country image of before coming to China; ACI, overall country image of after coming to China; PNI, positive national image; NNI, negative national image; EI, economic image; PI, product image.

Regression analysis was performed in the conditions of controlling the demographic variables (including citizenship, gender, age, educational degree, duration in China, and Chinese proficiency) to investigate the effects of CI on national image, product image, economic image, and residence intention after controlling for the effects of demographic variables. Table 7 shows that CI could significantly predict the product image (β = 0.28, *p* < 0.01), economic image (β = 0.27, *p* < 0.01), positive national image (β = 0.50, *p* < 0.01), negative national image (β = −0.29, *p* < 0.01), and residence intention (β = 0.43, *p* < 0.01).

**Table 7:** The results of the regression.

**Model**	**β**	***T***	**Δ*R^2^***
CI 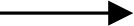 PI	0.28	3.51***	0.05
CI 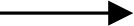 EI	0.27	4.14***	0.04
CI 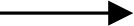 PNI	0.5	9.79***	0.23
CI 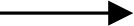 NNI	−0.29	5.04***	0.08
CI 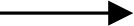 RI	0.43	6.80***	0.14

*p < 0.05.

**p < 0.01.

***p < 0.001.

CI, overall country image of after coming to China; PI, product image; EI, economic image; PNI, positive national image; NNI, negative national image; RI, resident intention; ΔR^2^, the explanatory rate of independent variables to dependent variables while demographic variables were controlled.

## Discussion

There is an increasing need for China to establish responsible country image and cooperative relationships with neighboring countries along the “One Belt and One Road.” In this context, this study aimed to build the theoretical foundation of China's CI and to provide a more comprehensive understanding of the conceptualization and operationalization of China's CI from the perspective of international students in Central Asian countries. Attracting more international students can not only allow other countries to gain a more accurate understanding of China, but also facilitate political and economic cooperation between countries and help China to enhance positive CI. International students studying in China can directly interact with Chinese people, which helps to reduce the cognitive bias and then form a more accurate understanding of China. Therefore, international students play important roles in helping other countries to construct an unbiased view of China's CI, and exchange study programs are essential in establishing stable and strong ties between countries.

First, the study made a comprehensive literature review and concluded the overall definition and conceptualization of CI. Then, we adapted an overall country image scale according to previous studies and examined the psychometric model of China's CI. Specifically, comparative analyses were used to investigate the four characteristics of CI such as integrity, multidimensionality, dynamics, and relative stability in the eyes of the international students from central Asian countries before and after their coming to China. Subsequent analyses examined CI's effects on product image, economic image, and citizen image; the analyses also provided additional insights on the relationships between these constructs.

Second, a 20-item scale has been developed to measure the overall CI of China. The CFA results reveal four interrelated dimensions, which can be loaded onto a single seconder-order construct representing the overall CI (Laroche et al., 2005; Wang et al., 2012). There are reasons to support the existence of a common underlying factor that might explain the correlations of the four factors. First, from a theoretical perspective, all items measuring the four dimensions also mirror the theoretical conceptualization of the overall CI. Second, from a methodological perspective, both the composite reliability and the homogeneity coefficient of the overall CI scale are greater than the recommended cutoff of 0.70 (Bagozzi and Yi, 1988). The results indicate that CI is a multidimensional construct composed of a cognitive dimension and an affective dimension. The cognitive dimension includes government image, social image, and international image. According to Nagashima (1970), the CI of a country is formed based on its national characteristics and its political, economic, and cultural background. The multidimensional nature of the CI construct is also supported by other researchers (e.g., Nagashima, 1970; Parameswaran and Yaprak, 1987; Gallarza et al., 2002). It is theoretically and practically important to develop a sound measurement scale of CIs, as it may provide a comprehensive understanding of the constitution of CI and its effects on people's behavior intentions, such as willingness to reside in a given country. Therefore, a valid measure of CI is essential for the development and implementation of cooperative strategies between countries.

Third, this study shows that international students from central Asian countries would develop a better impression of China's government image, international images, and overall CI after coming to China, whereas there is no significant change in social image. More specifically, central Asian students' perceptions and feelings of the leadership of government image, technology, education, military, films and TV, and transportation are greatly improved after coming to China. It is worth mentioning that the affective component of the overall CI increases most significantly. Thus, it is concluded that ones' beliefs and attitudes toward a country do not stay unchanged overtime, but vary with their direct or indirect contact with the country. However, no significant change is observed for items that evaluate urban hygiene and social security, and the evaluation of food safety and air quality is significantly decreased after coming to China, indicating that there is a need for China to improve food safety, urban hygiene, and air quality. These findings suggest that first-hand experience plays an important role in shaping one's perceptions and feelings toward a country.

Finally, and most importantly, this study contributes to broadening the understanding of the effects of CI on economic image, product image, positive and negative national image, and residence intention in China. The results demonstrate that CI can significantly predict these outcome variables, and thus, the higher the evaluations individuals hold toward the overall image of China, the more positive the perceptions and feelings of national image, product image, and economic image are, and they are more likely to reside in China. Therefore, the individual often associates the holistic belief of CI with the perception and evaluation of specific images such as product image, economic image, and national image. These provide strong support to the claim that a positive CI toward a country might benefit economic development, political stability, effectiveness and morality of their national and international policies, and the attractiveness of their culture (Werron, 2014).

However, the limitations of this study should also be noted. For example, only international students are recruited in this study, which may not provide a representative view of the whole population. Moreover, it is difficult to measure cultural differences that are rooted in history, religion, education, values, and attitudes. Despite these limitations, the findings of this study provide valuable strategic information to policymakers. It is important for policymakers as well as public and private organizations to be aware of the power of CI in achieving national goals.

## Data availability statement

The raw data supporting the conclusions of this article will be made available by the authors, without undue reservation.

## Ethics statement

The studies involving human participants were reviewed and approved by the Research Ethics Committee of Shaanxi Normal University approved the study. Prior to testing, we obtained both written and informed consent from all participants. Written informed consent for participation was not required for this study in accordance with the national legislation and the institutional requirements.

## Author contributions

XY was mainly responsible for the supervision and guidance of the entire research process, timely correct the wrong ideas and direction, and played an important role in the successful writing of the paper. XZ was responsible for the research plan of this research, for example, to determine the research object, clear research methods, contact the subjects to test, data entry, and so on, and finally to collect the data back analysis to verify the assumptions made by this study, and SL is responsible for the study of the full text of the writing work. All authors contributed to the article and approved the submitted version.

## Funding

This research was supported by “Belt and Road Initiative” Institute of Development (No: YDYL2020YB015) and Development Research Center of China' Xinjiang Cooperative with Neighboring Countries in Yili Normal University (No: 2021ZBGJZD001).

## Conflict of interest

The authors declare that the research was conducted in the absence of any commercial or financial relationships that could be construed as a potential conflict of interest.

## Publisher's note

All claims expressed in this article are solely those of the authors and do not necessarily represent those of their affiliated organizations, or those of the publisher, the editors and the reviewers. Any product that may be evaluated in this article, or claim that may be made by its manufacturer, is not guaranteed or endorsed by the publisher.
